# Improvements in High Resolution Laryngeal Magnetic Resonance Imaging for Preoperative Transoral Laser Microsurgery and Radiotherapy Considerations in Early Lesions

**DOI:** 10.3389/fonc.2018.00216

**Published:** 2018-06-06

**Authors:** Thomas Ruytenberg, Berit M. Verbist, Jordi Vonk-Van Oosten, Eleftheria Astreinidou, Elisabeth V. Sjögren, Andrew G. Webb

**Affiliations:** ^1^C.J. Gorter Center for High Field MRI, Department of Radiology, Leiden University Medical Center, Leiden, Netherlands; ^2^Department of Radiology, Leiden University Medical Center, Leiden, Netherlands; ^3^Department of Radiotherapy, Leiden University Medical Center, Leiden, Netherlands; ^4^Department of ENT – Head and Neck Surgery, Leiden University Medical Center, Leiden, Netherlands

**Keywords:** glottic carcinoma, transoral laser microsurgery, magnetic resonance imaging, laryngeal imaging, radiofrequency coil arrays, imaging protocols

## Abstract

As the benefits, limitations, and contraindications of transoral laser microsurgery (TLM) in glottic carcinoma treatments become better defined, pretreatment imaging has become more important to assess the case-specific suitability of TLM and to predict functional outcomes both for treatment consideration and patient counseling. Magnetic resonance imaging (MRI) is the preferred modality to image such laryngeal tumors, even though imaging the larynx using MRI can be difficult. The first challenge is that there are no commercial radiofrequency (RF) coils that are specifically designed for imaging the larynx, and performance in terms of coverage and signal-to-noise ratio is compromised using general-purpose RF coils. Second, motion in the neck region induced by breathing, swallowing, and vessel pulsation can induce severe image artifacts, sometimes rendering the images unusable. In this paper, we design a dedicated RF coil array, which allows high quality high-resolution imaging of the larynx. In addition, we show that introducing respiratory-triggered acquisition improves the diagnostic quality of the images by minimizing breathing and swallowing artifacts. Together, these developments enable robust, essentially artifact-free images of the full larynx with an isotropic resolution of 1 mm to be acquired within a few minutes.

## Introduction

Early as well as moderately advanced laryngeal cancers are both highly treatable conditions, with the main treatment options consisting of transoral laser microsurgery (TLM), open partial laryngectomy (OPL), and radiotherapy (RT). All three modalities are used both in primary as well as recurrent disease, and treatment choice depends on the extent of the lesion as well as local therapeutic protocols and patient-specific factors such as age, comorbidity, and patient preference. Advances in therapy over the past two decades have enabled the relative benefits and limitations of the various treatment methods to be better understood. In particular, the indications for TLM have been more clearly defined (1).

The benefits of TLM include the low morbidity and short treatment time associated with endoscopic removal of the tumor. Functional outcomes are better than after OPL (2) and, for early lesions, they are comparable to RT (3). In moderately advanced lesions, there is as of yet a lack of comparative evidence between TLM and RT concerning functional outcome to draw any definite conclusions (4, 5). Finally, as TLM leaves all treatment options open after treatment, larynx preservation is generally higher than after RT (5–7). However, as TLM has evolved, and moderately advanced tumors are increasingly being treated, tumor extension to certain subsites within the larynx have been associated with a higher risk of recurrence after TLM (1). This has put an increasing requirement on high-quality radiological imaging to select those cases most suited for a transoral approach as opposed to open surgery or RT.

Imaging has become crucial for accurate evaluation, not only of tumor localization and size, but also for the involvement of subsites for which the suitability of TLM is still under debate, such as the pre- and posterior paraglottic space, the cricothyroid membrane, and the inner lamina of the thyroid cartilage (1). Magnetic resonance imaging (MRI) is increasingly used to distinguish between tumor and edema or fibrosis (8). In addition, in tumors in which surgery is indicated, imaging can help delineate tumor borders and predict the extent of the resection needed. This information can then be used in preoperative patient counseling regarding functional results, which are related to the extent of the resection. Finally, as the role of TLM expands, high quality imaging has become increasingly important in postoperative follow-up for early detection of submucosal recurrence, therefore, maintaining the possibility to perform open partial salvage surgery (1) or administering salvage RT. As a result, as treatment selection, patient counseling and follow-up monitoring for primary and recurrent early and moderately advanced laryngeal cancer evolves, the demand for high quality, robust, high resolution imaging has increased accordingly.

In tumors where RT is chosen as the preferred treatment of a glottic cancer, high-resolution imaging plays an equally important role. The goal of RT is to deliver a high dose to the tumor and minimize the dose to the surrounding healthy tissues in order to reduce vocal dysfunction, dysphagia, arytenoid edema, and carotid artery injuries (9). This goal can only be achieved if the tumor is defined with high accuracy, i.e., when high resolution imaging is available.

**Figure 1:**
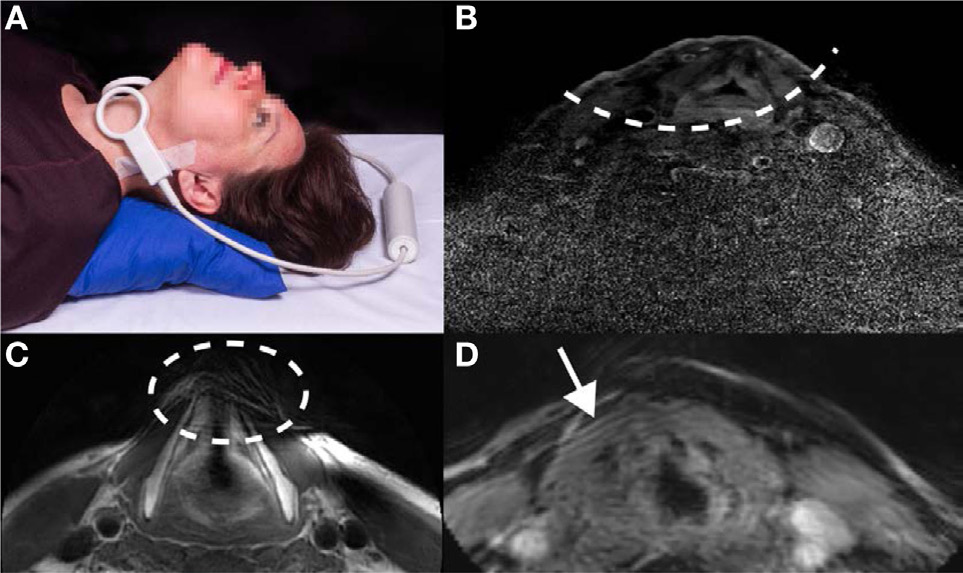
Illustrations of the challenges associated with current approaches to laryngeal magnetic resonance imaging. **(A)** Two individual, commercial circular receive coils, each with a diameter of 4 cm, are placed around the larynx. Positioning in this particular setup is critical for imaging the larynx and can be experienced as uncomfortable by the subject since the loops have to be held firmly in place with tape and/or velcro straps. Written informed consent was obtained from the subject for publication of this photo. **(B)** Images acquired with a limited penetration depth due to incorrect coil positioning, **(C)** images showing signal voids in the anterior part of the larynx due to incorrect coil positioning, and **(D)** images showing movement artifacts due to subject breathing.

Historically, computed tomography (CT) has been the imaging modality of choice, particularly for RT (10). CT is a robust modality due to very short acquisition times and high signal-to-noise ratio (SNR), but is limited in its utility since differentiation between healthy tissue and tumors can be difficult (10, 11). Imaging the larynx using MRI became a useful alternative to CT in the late 1980s and early 1990s primarily due to the development of local surface coils, which enabled higher resolution imaging over a field-of-view, which could be localized to the larynx (12–15). The lack of ionizing radiation, greater inter-tissue contrast, and multiplanar imaging capabilities were all advantages of MRI over CT, but long acquisition times limited the spatial resolution that could be achieved. Only when parallel imaging became available to reduce MRI acquisition times was it possible to achieve high spatial resolution in a realistic scanning time (16). However, laryngeal imaging is still problematic. Current MRI protocols in early glottis carcinoma primarily use general purpose commercial local surface coils (which can also be combined with a head coil), or phased array head/neck coils (8, 17–23). As these coils (for example two loops as shown in Figure 1A) are not specifically designed for laryngeal imaging, correct positioning centered on the vocal cords is critical, and needs to be done carefully and precisely by an experienced technician. Problems also arise with different-sized necks, which require a different physical separation of the coils. If the coils are placed too close together then they couple to one another, reducing the signal-to-noise, and also result in too small a field-of-view, a reduced penetration depth, and poor parallel imaging performance (including the possibility of fold-back artifacts): a typical example is shown in Figure 1B. If the loops are placed too far apart, or the patient has a large neck, signal voids within the larynx may occur, as shown in Figure 1C. In addition, the separate loops are prone to displacement during the examination, which increases the patient handling time and may induce patient discomfort.

Even if proper positioning of the coil has been performed, reconstructed images of the larynx often suffer from patient-related artifacts, which in some cases render images non-useful for diagnosis. One common cause is movement of the larynx during data acquisition, as shown in Figure 1D. Movement is unavoidable during the scanning protocol, as breathing causes the vocal cords to move. Swallowing, which moves the full larynx up to several centimeters in the feet–head direction, may occur and can produce severe image artifacts. In addition, pulsation in the blood vessels in the neck commonly creates flow artifacts, which can extend into the larynx region, depending on the imaging protocol used.

In this paper, we report on our work to improve the robustness and performance of high resolution laryngeal MRI. This consisted of two different aspects: first the design, construction, and testing of a flexible receive-array coil, which can adapt to different neck sizes, be worn comfortably by the patient, and enable robust parallel imaging for reduced scanning time. In addition, the array can be used for patients who have to be scanned in a RT fixation mask and it is, to our knowledge, the first time that a coil has been specifically designed to fit such fixation devices. In parallel, we investigated the use of different scan protocols to reduce image artifacts associated with patient motion and pulsatile blood flow.

## Materials and Methods

### Coil Development

**Figure 2:**
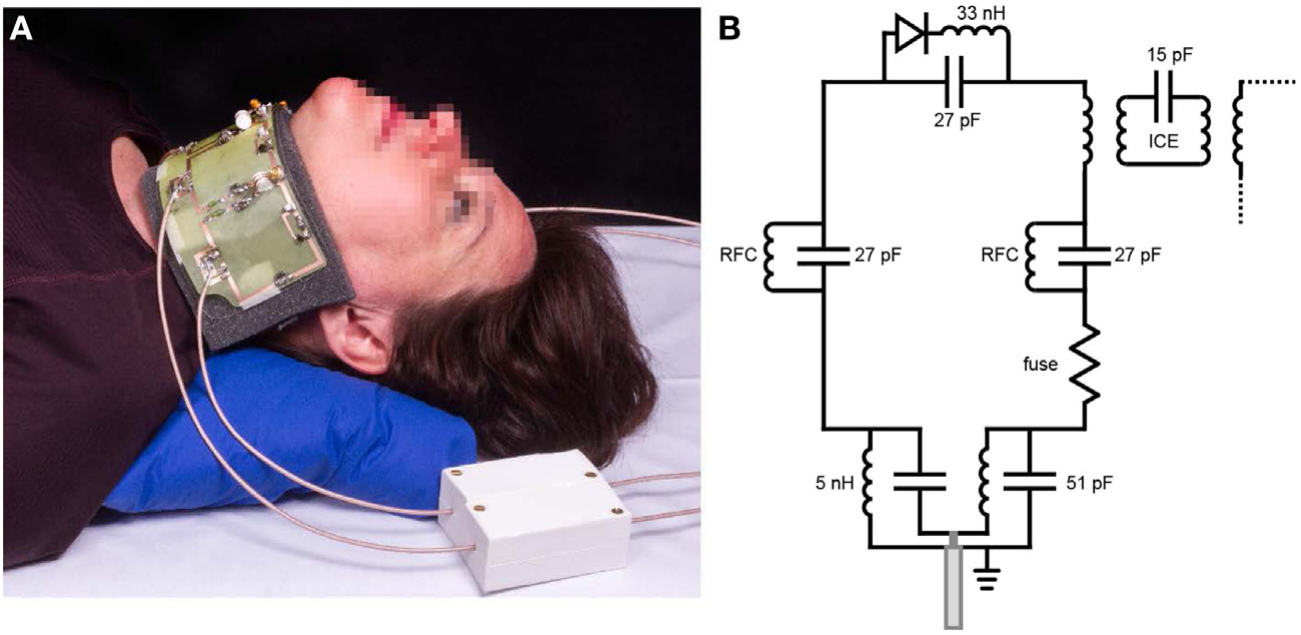
**(A)** The dedicated larynx coil with the electronics visible (an encased version is used for *in vivo* scans). The coil is flexible and, therefore, adjustable to different neck sizes. The white boxes are floating cable traps, which prevent current from flowing on the outer shield of the connecting cables. Written informed consent was obtained from the subject for publication of this photo. **(B)** A schematic of the electrical circuit of each of the four loops constituting the coil. The individual loops are decoupled from the neighboring elements using an induced current elimination (ICE) circuit. Radiofrequency chokes (RFCs) are used to allow a DC current path to turn on the PIN diodes to isolate the receive coils from the transmit body coil.

The dedicated radiofrequency (RF) coil receive array was designed to be flexible to accommodate different neck sizes and to have a high degree of isolation between individual elements to allow high acceleration factors while maintaining a high SNR (16, 24). The number of coils in the receive array represents a compromise: a large number enables a high degree of acceleration, but increases the complexity of the networks required to decouple each of the coils and reduces the flexibility of the overall structure. In order to maintain a high degree of flexibility, as well as maintaining a high degree of decoupling under the different flexation angles required by different-sized necks, we constructed a one-dimensional four element array, as shown in Figure 2.

The dedicated coil is a four-element receive-only array and consists of four identical loops (60 mm × 60 mm each). Inter-element decoupling is achieved using a method termed induced current elimination (25) in combination with preamplifier decoupling, which uses a low impedance pre-amplifier acting as a current transformer (24). This combination results in a coupling of less than 0.1% for neighboring elements and 1% for non-neighboring elements. This high level of decoupling is necessary to enable efficient and artifact-free parallel imaging performance (16). Each coil is tuned at 128 MHz (3 T) and impedance matched to 50 Ω using a lattice balun, which also suppresses common mode currents. Further common mode suppression was achieved by using floating cable traps (26). Decoupling from the transmit body coil (detuning) is performed by a single active PIN diode trap in the each of the resonant loops. A fuse (250 mA very fast acting fuse; Littlefuse 0251.250MXL) is integrated into every loop to provide intrinsic safety in the case of any failure in detuning by disabling the coil in case of abnormal high currents.

The printed circuit board of the coil is etched on 0.125 mm thick FR4, which is flexible enough to be mounted on a curved 1 mm thick acrylic sheet to provide robustness to the coil as well as flexibility. The geometry of the coil is chosen to fit closely but comfortably around the neck and due to the flexibility of the coil, it easily accommodates different neck sizes. This also opens the possibility for the coil to fit on a RT mask, used to secure the head and neck during RT treatment.

### Protocol Development

Magnetic resonance imaging scans were performed using a 3 T Philips Ingenia system, with the scan parameters shown in Table 1. *In vivo* scans were first performed on healthy volunteers. The local medical ethics committee approved all studies, and informed consents were obtained from all volunteers prior to the MR scan.

**Table 1:** Scan parameters used for volunteer and patient studies.

	T1-weighted TSE	T1-weighted TSE (with SPIR fat suppression)	T2-weighted Dixon TSE	T2-weighted Dixon TSE	DWI
TE/TR (ms)	5.9/725	5.9/649	100/4680	60/2410	51/2038
Scan plane	Transversal	Transversal	Transversal	Coronal	Transversal
Voxel size (mm^3^)	1.0 × 1.0 × 1.0	1.0 × 1.0 × 1.0	1.0 × 1.0 × 1.0	0.65 × 0.81 × 1.5	3.0 × 3.0 × 3.0
Field of view (mm^3^)	200 × 200 × 40	200 × 200 × 40	200 × 180 × 40	200 × 200 × 60	108 × 280 × 198
Phase direction	Anterior–posterior	Anterior–posterior	Anterior–posterior	Right–left	Anterior–posterior
Acceleration factor	3.0	3.0	1.5^b^	2.3	3.0
Averages	1	1	2	1	1
Total duration^a^ (seconds)	189	216	253	54	168

*^a^As all scans are respiratory triggered, scan time is dependent on the breathing pattern of the subject and the times shown are based on a breathing rate of once every 7 s. TSE, turbo-spin echo; DWI, diffusion-weighted imaging; SPIR, spectral presaturation with inversion recovery*.

*^b^An acceleration factor of 1.5 was used during the scans of the two patient cases described in the results section of this paper. Since that time, the protocol has been changed such that all transversal T2-weigthed Dixon TSE scans are performed using acceleration factors of 3.0, reducing scan time to 126 s*.

The four-channel phased array receive coil is interfaced to the scanner through a dedicated conversion box. During all scans, the commercial posterior array coil, which is built into the patient bed was electronically disconnected. In order to assess the performance of the receive coil array, scans on a cylindrical saline phantom (12 cm diameter) representing the neck were performed. The maximum acceleration factor that results in artifact-free images was determined from these phantom scans. Acceleration factors of between 2 and 4 in either the anterior–posterior or right–left directions showed that fold-over artifacts at a factor of three are present but do not fold-in over the region of the larynx.

In order to address movement artifacts, respiratory-triggered scans were implemented, meaning that images are only obtained during a limited portion of the breathing cycle (in this case during exhaling), ensuring a stable position of the larynx during data acquisition. The respiratory trigger was applied using a belt on the abdomen, which triggered 500 ms after the initial stage of exhalation. Interestingly, since breathing and swallowing are physiologically linked (swallowing often occurs after exhaling), the use of respiratory-triggered scans also prevents swallowing-induced artifacts during image acquisition.

With respect to reducing image artifacts due to pulsatile blood flow in the arteries, we determined that implementing spatial saturation pulses caudal to the larynx did not result in a significant decrease in pulsation artifacts, and so were not used. Instead, to prevent pulsation artifacts falling within the larynx, the phase encoding direction was chosen to be in the anterior–posterior direction (23) for all transversal scans. For coronal scans, the anterior–posterior direction represents the slice-select direction for two-dimensional scans. The feet–head direction would be preferred for these scans, however, due to the geometry of the RF coil array acceleration cannot be obtained in this phase encoding direction. Right–left phase encoding is, therefore, the only possible phase encoding direction for coronal slices. This can sometimes result in pulsatile artifacts in the posterior part of the larynx.

## Results

### Healthy Volunteers

**Figure 3:**
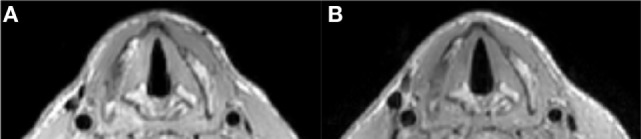
Respiratory triggered T1 TSE sequence (1 mm isotropic voxel size) with parameters as in Table 1. **(A)** Shows an image at the level of the vocal cords made with the two loops setup (as in Figure 1A) and at the maximum acceleration factor of 2.0: total scan time 234 s. **(B)** Shows an image at the same level made with the dedicated coil and an acceleration factor of 3.0: total scan time 189 s.

A comparison between the setups shown in Figure 1A (two commercial loops) and Figure 2A (dedicated coil) is shown in Figure 3. The scan with the loops placed on a cooperative volunteer was acquired with an acceleration factor of two (the maximum achievable factor for a two-element coil), while the scan with the dedicated coil was performed with an acceleration factor of three. The image quality between the two setups is very similar (when the two commercial loops are perfectly positioned), but the scan with the dedicated coil could be performed with a 33% reduction in scan time. The images show that the dedicated coil outperforms the commercially available setup even under ideal scanning conditions.

**Figure 4:**
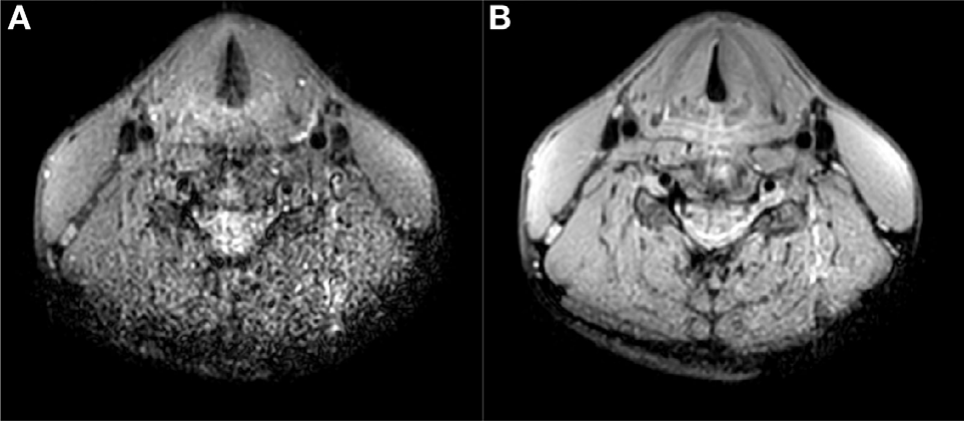
T1 TSE multi-slice sequence (transversal) at 1.0 mm isotropic resolution with SPIR fat suppression (scan parameters in Table 1) **(A)** without respiratory trigger, duration 57 s, **(B)** with respiratory trigger, duration 216 s. Triggering mitigates breathing and swallowing motion artifacts and increases image quality.

Figure 4 shows a comparison of images acquired with and without respiratory triggering. Respiratory triggering obviously increases the total imaging time, but the acceleration factor of three using the dedicated coil array reduces the scan time to 3 min and results in images without breathing and swallowing motion artifacts while at the same time also improving general image quality.

**Figure 5:**
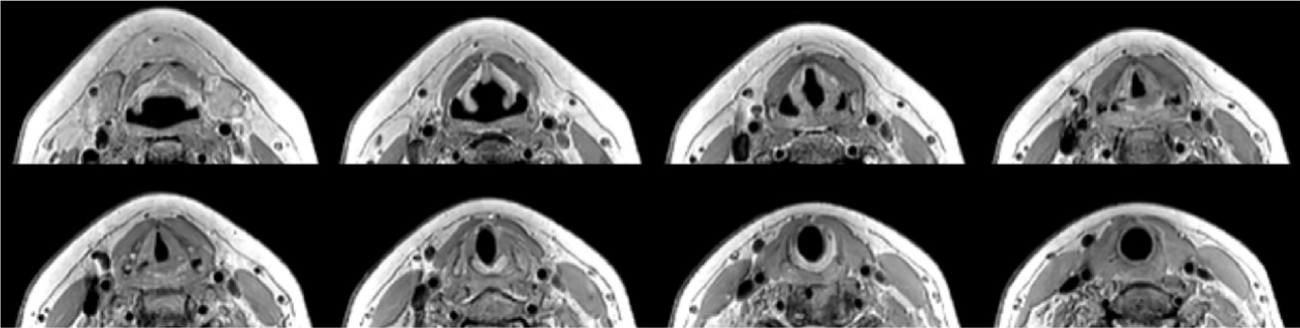
T1 TSE 1.0 mm isotropic scan with parameters as in Table 1. A selection of eight out of 40 slices ranging from aryepiglottic fold to subglottis on a healthy volunteer showing depth of penetration far beyond the larynx.

With the dedicated larynx coil, the entire length of the larynx can be covered and also the penetration depth is more than sufficient for imaging the full larynx. In total, 11 volunteers have been scanned with this setup, showing similar image quality for all volunteers. An example of the field of view is shown is shown in Figure 5.

### Patient Scanning

After scanning healthy volunteers for protocol optimization, scans were performed on two patients with suspected tumor recurrence after treatment. The full scan protocol for patients includes a transversal T1 TSE, transversal and coronal T2 TSE Dixon, transversal DWI sequence before gadolinium being administered and a set of transversal T1 TSE scans with SPIR (spectral presaturation with inversion recovery) fat suppression after gadolinium administration. This protocol takes less than 30 min.

#### Patient Case 1

**Figure 6:**
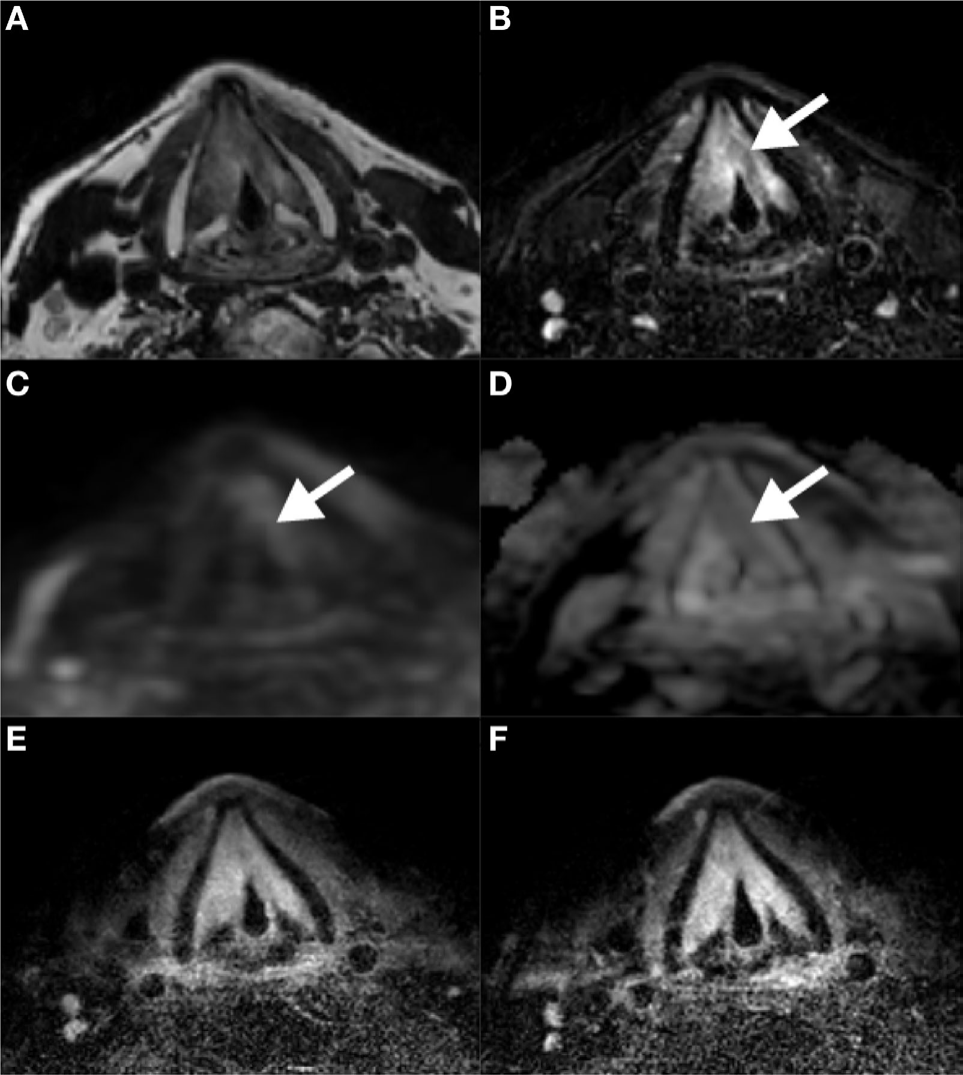
Magnetic resonance imaging scans 8 months after radiation therapy for a T1b glottis carcinoma. Increased signal intensity on T2-weighted scans without **(A)** and with **(B)** fat suppression in both vocal cords, together with the absence of diffusion restriction on DWI/ADC **(C,D)** is compatible with posttreatment edema. A biopsy in the superficial area corresponding to intermediate signal on T2 and diffusion restriction on the left vocal cord revealed tumor recurrence. Contrast-enhanced T1-weighted scans at two different time points show gradual enhancement **(E,F)**.

A 64-year-old male patient presented with a carcinoma of the left vocal cord with involvement of the anterior commissure and the anterior part of the contralateral vocal cord (T1b). Because of limited endoscopic exposure of the larynx, TLM could not be performed and the patient was treated with RT (25 fractions of 2.4 Gy each). Due to anxiety disorder, the patient refused flexible laryngoscopy during follow-up. For further surveillance, an MRI examination of the larynx was requested 8 months after irradiation. A subsection of the images acquired using the new coil and imaging protocols are shown in Figure 6. On T2-weighted images without and with fat suppression increased signal intensity was seen in both vocal cords and an area with intermediate signal on the surface of the left vocal cord (Figures 6A,B). On DWI, the T2 hyperintense areas showed no diffusion restriction, corresponding to inflammatory edema. DWI showed diffusion restriction in the superficial portion of the left vocal cord, compatible with submucosal recurrence (Figures 6C,D). In this patient, slowly increasing enhancement was seen and in tumor recurrence enhancement has been reported to be variable (8) (Figures 6E,F). Histopathological examination of biopsy specimens from the left vocal cord confirmed the presence of squamous cell carcinoma.

#### Patient Case 2

**Figure 7:**
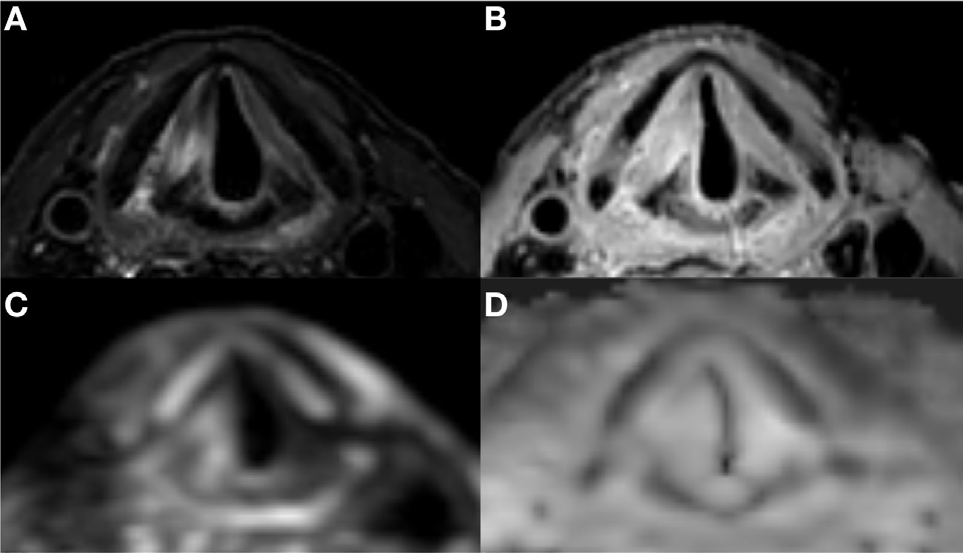
Laryngeal magnetic resonance imaging of a patient with superficial recurrence after radiotherapy of an early glottis carcinoma: high signal intensity in the right vocal cord and posterior paraglottic space on T2 **(A)** combined with a high signal intensity on both DWI **(C)** and ADC **(D)** is compatible with post irradiation edema and rules out deep tumor extension. There is diffuse enhancement on contrast enhanced T1 with fat suppression **(B)**.

During follow-up of an 82-year-old male patient, who underwent RT for a T2 glottic carcinoma of the right vocal cord 1 year previously with sub- and supraglottic extension, an ulceration suggestive of tumor recurrence was detected. The patient was referred for imaging to evaluate the depth of extension. Figure 7 shows four scans used for assessment using the new receive coil array and respiratory-triggered scans. Overall, the scans revealed a paramedian position of the right vocal cord and widening of the space between the thyroid cartilage and arytenoid. On the T2-weighted images, hyperintense signal is seen in the posterior portion of the vocal cord and in the posterior paraglottic space. This area showed enhancement, but no diffusion restriction. This MRI pattern is compatible with inflammatory changes or edema secondary to radiation therapy. Examination under anesthesia was performed and a biopsy of the superficial ulceration was taken. The underlying thyroarytenoid muscle appeared free of tumor. Pathologic exam confirmed a superficial tumor recurrence.

## Discussion

Early and moderately advanced laryngeal carcinoma are well treatable diseases by the main treatment modalities of TLM, OPL, and RT, all of which have their relative benefits and limitations. In choosing and administering the most suitable treatment option for the individual patient, there are increasing demands for high quality imaging to: (1) assess image-based factors that determine the relative suitability for the different treatment modalities, (2) distinguish edema from tumor tissue, (3) predict the extent of the resection needed and the functional consequences, (4) identify early submucosal recurrence, and (5) improve tumor delineation in highly focused RT strategies. Key factors to obtain these high quality, artifact-free MR images of the glottis are to have a coil with high local sensitivity and to perform data acquisition with protocols, which reduce any motion-induced imaging artifacts. Reviewing previous literature, most groups use commercial general purpose surface coils. 3D gradient echo T1 sequences with fat suppression (for example, volumetric interpolated breath-hold examination) can be obtained with submillimeter voxels in cooperative patients (8, 18, 20). For TSE sequences, slice thicknesses of 3–5 mm are chosen to reduce noise and acquisition time (11, 17, 18, 20–23).

Our main aim in this work is to improve on these previously reported scan protocols, particularly with respect to reduced slice thickness in TSE sequences. In order to produce artifact-free images, respiratory-triggered sequences were employed, which intrinsically increases the image acquisition time compared to non-triggered sequences. In order to at least partially compensate for this, an RF coil with high parallel imaging performance needed to be designed, which can be achieved by fabricating a coil with multiple individual elements. Many design criteria had to be taken into account and multiple prototypes have been built. The coil had to be flexible and fit many different neck-sizes, while being robust to ensure that soldering of the electronic components was not compromised under flexation. With the final design consisting of four channels, it is able to scan fast with an acceleration factor of three and allows for imaging with adequate SNR using a slice thickness of only 1 mm and a voxel size of 1 mm^3^.

Having obtained considerable experience in scanning both healthy volunteers and patients using the dedicated larynx coil and respiratory-triggered acquisitions, we anticipate that many aspects of image acquisition are worth pursuing to improve the efficiency of data acquisition. For example, we have seen that some sequences do not fully use the available acquisition time during the exhalation. This unnecessarily lengthens these scans by requiring more breathing cycles for data acquisition. Other methods to obtain faster scans, other than parallel imaging, such as compressed sensing (27) have not yet been implemented and can result in substantial scan duration reduction.

In addition, it should be noted that the use of a respiratory trigger also creates extra constraints and challenges. For example, it requires the subject to have a regular breathing pattern that is not too slow, as a slow breathing pattern will slow down acquisition and lengthen the scans. Furthermore, when performing dynamic contrast enhanced scans, due to the lengthening of the scans with the respiratory trigger, early contrast enhancement may be missed. These scans should, therefore, not be performed using a trigger. A last point is the fact that we have initially chosen the 1.0 mm isotropic resolution for most scans in order to be able to easily reslice in any given direction. This reslicing has not always been successful, as very slight displacement between the slices is observed originating from some residual motion, resulting in staircasing of anatomical boundaries when performing multi-slice 2D acquisitions. For improving the performance of coronal scans, a coil with a higher spatial encoding power in the feet–head direction would be beneficial in order to obtain a better acceleration performance.

The dedicated coil has also been designed with integration with RT in mind. Patients receiving RT for laryngeal tumors are fixated in a customized individual mask. Fixation of the patient ensures treatment delivery reproducibility, as RT is delivered not in one but in many separate fractions. Because of the required reproducibility, the CT treatment planning is also done in a mask, as should be any diagnostic MR for RT purposes to allow for accurate image registration. In this way, the advantages of tumor delineation for RT based on a high-resolution MRI are fully utilized (28). Accurate tumor delineation is the first step of the RT process for a highly focused dose delivery, which is critical when aiming for voice preservation and less side effects such as dysphagia, xerostomia, or stroke related to the dose received by the carotid arteries. Our coil is the first dedicated laryngeal coil, to our knowledge, which has been designed to fit a RT mask (23).

## Conclusion

The developed dedicated larynx coil and scan protocols allow for high resolution imaging (1.0 mm isotropic for TSE sequences) of the larynx, without being affected by breathing or swallowing artifacts. The coil allows efficient parallel imaging, which is used to speed up data acquisition. Furthermore, the coil is flexible and, therefore, easily accommodates different neck sizes. Additionally, the short total protocol time for patients (30 min maximum) and the fact that they can continue breathing and swallowing during the scans, reduces the burden of these scans for patients. We anticipate that this improved image quality will lead to better treatment planning and counseling in patients with laryngeal tumors.

## Data Availability Statement

Datasets are available on request: the raw (anonymized) data supporting the conclusions of this manuscript will be made available by the authors, without undue reservation, to any qualified researcher.

## Ethics Statement

This study was carried out in accordance with the recommendations of the Medical Ethics Committee of the Leiden University Medical Center. The protocol was approved by the Medical Ethics Committee of the Leiden University Medical Center. All subjects gave written informed consent in accordance with the Declaration of Helsinki.

## Author Contributions

TR built the MR coil. TR, BV, and JO developed the MR scan protocols and performed the scans. TR, BV, ES, EA, and AW have written the manuscript. All authors contributed to the scientific debate.

## Conflict of Interest Statement

All authors declare that the research was conducted in the absence of any commercial or financial relationships that could be construed as a potential conflict of interest.
